# The Dark Recovery Rate in the Photocycle of the Bacterial Photoreceptor YtvA Is Affected by the Cellular Environment and by Hydration

**DOI:** 10.1371/journal.pone.0107489

**Published:** 2014-09-11

**Authors:** Francesca Pennacchietti, Stefania Abbruzzetti, Aba Losi, Carmen Mandalari, Roberta Bedotti, Cristiano Viappiani, Francesca Cella Zanacchi, Alberto Diaspro, Wolfgang Gärtner

**Affiliations:** 1 Fondazione Istituto Italiano di Tecnologia, Genova, Italy; 2 Dipartimento di Fisica e Scienze della Terra, Università di Parma, Parma, Italy; 3 NEST, Istituto Nanoscienze, Consiglio Nazionale delle Ricerche, Pisa, Italy; 4 Max-Planck-Institute for Chemical Energy Conversion (CEC), Mülheim a.d. Ruhr, Germany; Tokai University, Japan

## Abstract

We report thermal recovery kinetics of the lit state into the parental dark state, measured for the blue light-sensing photoreceptor YtvA inside overexpressing *E. coli* and *B. subtilis* bacterial cells, performed for the wild type and several mutated proteins. Recovery was followed as a recovery of the fluorescence, as this property is only found for the parental but not for the photochemically generated lit state. When cells were deposited onto a microscope glass plate, the observed thermal recovery rate in the photocycle was found ca. ten times faster in comparison to purified YtvA in solution. When the *E. coli* or *B. subtilis* colonies were soaked in an isotonic buffer, the dark relaxation became again much slower and was very similar to that observed for YtvA in solution. The observed effects show that rate constants can be tuned by the cellular environment through factors such as hydration.

## Introduction

YtvA is a blue light photoreceptor from *Bacillus subtilis*, composed of an N-terminal flavin-binding LOV- (light, oxygen, voltage) domain, sharing high structural and sequence homology with the flavin mononucleotide (FMN)-binding LOV domains of plant phototropins (phot), [1] and a C-terminal STAS domain (sulphate transporter anti sigma factor antagonist). In *B. subtilis*, YtvA is part of the stress response complex that is activated under threatening environmental conditions. [2] When the dark adapted species (YtvAD) is illuminated with blue light, a photocycle is initiated, which proceeds through a triplet state leading in high yield (0.49 [3]) to reversible formation of a blue-shifted FMN-cysteine C(4a)-thiol adduct (YtvAL). YtvAL is considered to be the biologically active, signalling state. The photoadduct slowly reverts in the dark to the parental state YtvAD with lifetime ca. 4500 s at 25°C. [4] This dark relaxation of YtvAL is a thermally activated process with a substantial enthalpic barrier (102 kJ M^−1^) and a favorable entropic barrier (31 J K^−1^ M^−1^). Whereas the parental state shows a notable fluorescence (Φ_fl_ = 0.22), the photoadduct state has lost the fluorescence capacity entirely.

Photochromicity between two moderately or fully stable states is an intriguing property of many photoreceptors as it implies potential employment of such proteins in biotechnological applications. In that respect, we could recently demonstrate that besides the thermally driven process, illumination of YtvAL with violet or near UV light results in population of YtvAD in appreciable yield (ca. 0.05). [5] The possibility of switching YtvA between the fluorescent YtvAD state and the non-fluorescent YtvAL state with light of different wavelength opens a variety of applications including super-resolution microscopy with random activation of fluorophores and bidirectional control of the photosensitive actuator [6], [7], [8].

Clearly, the rate constant for dark relaxation of the photoadduct YtvAL is a parameter of fundamental relevance both for fluorescence imaging studies and for optogenetic applications exploiting the photoswitchable chromophore as a bidirectional actuator.

However, in view of foreseen applications in cells it appears important to estimate the effect of cellular environment on photocycle rate constants, in particular, as experiments have revealed an extended hydrogen bonding network existing between the flavin chromophore and the surrounding amino acids that over a wide range modulates the thermal recovery kinetics. [9], [10] Moreover, the level of hydration has been shown to affect the latter kinetics in the LOV2 domain of *Avena sativa* phot1 (referred to as *As*LOV2). [11] We have thus measured dark recovery kinetics of YtvA in its natural host, *B. subtilis*, and in *E. coli* colonies overexpressing YtvA under different humidity level for the wild type protein (wt) and some mutants that had been identified to be instrumental for the dark recovery process.

## Methods

### E. coli and B. subtilis cultures

Heterologous expression of wt and mutated YtvA in *E. coli* was performed as described. [10] Proteins in solution used for these experiments were affinity purified from overexpressing *E. coli* cells employing a His6-tag at their C-terminal end. [10] *B. subtilis* cells overexpressing YtvA, were generated from an YtvA deletion mutant, transformed with an YtvA-encoding plasmid (U. Krauss, personal communication), and were kindly donated by Ulrich Krauss (FZ Jülich, Germany).

### Fluorescence emission by E. coli colonies

The fluorescence emission by *E. coli* or *B. subtilis* colonies over-expressing YtvA was collected through an upright epifluorescence microscope (Nikon Eclipse E600) equipped with a 10X long working distance objective (Plan Fluor10X/0.30 DIC L WD 16.0). A 150 W Xe arc lamp (LOT-Oriel) was used as excitation source. The white light intensity was modulated with a mechanical chopper (EG&G PARC 192) and entered into the epifluorescence excitation port through a fiber bundle. A filter cube allowed to excite the fluorescence emission by 390 nm light (Thorlabs MF390-18 excitation filter, Thorlabs MD416 Dichroic Filter) and collect fluorescence emission by YtvA through a suitable emission filter (Thorlabs MF525-39). The intensity modulated wide field fluorescence emission (collected over a ∼1 mm diameter area) was detected by a single channel photometer (model 814, PTI). The voltage output was fed into a dual channel lock-in amplifier (EG&G PARC, 5208) which allowed to efficiently reject the noise. The retrieved DC amplitude was recorded on a computer using a signal acquisition board (National Instruments 6013). Finally, actinic beams at 465 nm (LED456) and 405 nm (LED405) were entered through the bottom port of the microscope (see reference [5] for spectral characteristics). Maximum available power on the focal plane of LED465 and LED405 were 84 µW and 8 µW, respectively.

*E. coli* colonies were transferred from the Petri dish to a glass plate. For studies on *E. coli* colonies soaked in a buffered solution, colonies were transferred to an 8-well plate with volume 500 µl (Lab-Tek), then covered with 200 µL of a 10 mM phosphate buffer solution, containing 0.9% NaCl (W/V), pH = 7.4. Samples were then transferred to the microscope and kept in the dark for 1 hour prior to performing experiments.

#### FPALM microscopy

FPALM imaging was performed on a super-resolution microscope NIKON N-STORM equipped with a 100X 1.40 NA Nikon objective lend and an Andor Ixon DU-897E-CS0BV running at approximately 30 Hz (30 ms exposure time). The excitation scheme consisted of an activation laser at 405 nm (Coherent CUBE 405–100 mW) and a readout laser at 488 nm (Coherent Sapphire OPSL 488 nm-50 mW). Specific dichroic mirrors (Chroma, T505LP) and band-pass dichroic filters allowed selection of the emitted signal (Semrock BLP01-488R-25).

The molecules position is found, after background subtraction and thresholding, by means of a gaussian fitting procedure. The rendering of the super-resolution image is obtained plotting the position of each single event as a gaussian spot with standard deviation corresponding to the calculated localization precision. Before the rendering of the final image, a filter on brightness and molecule dimension is applied and unsuitable events are rejected [7].

## Results

**Figure 1:**
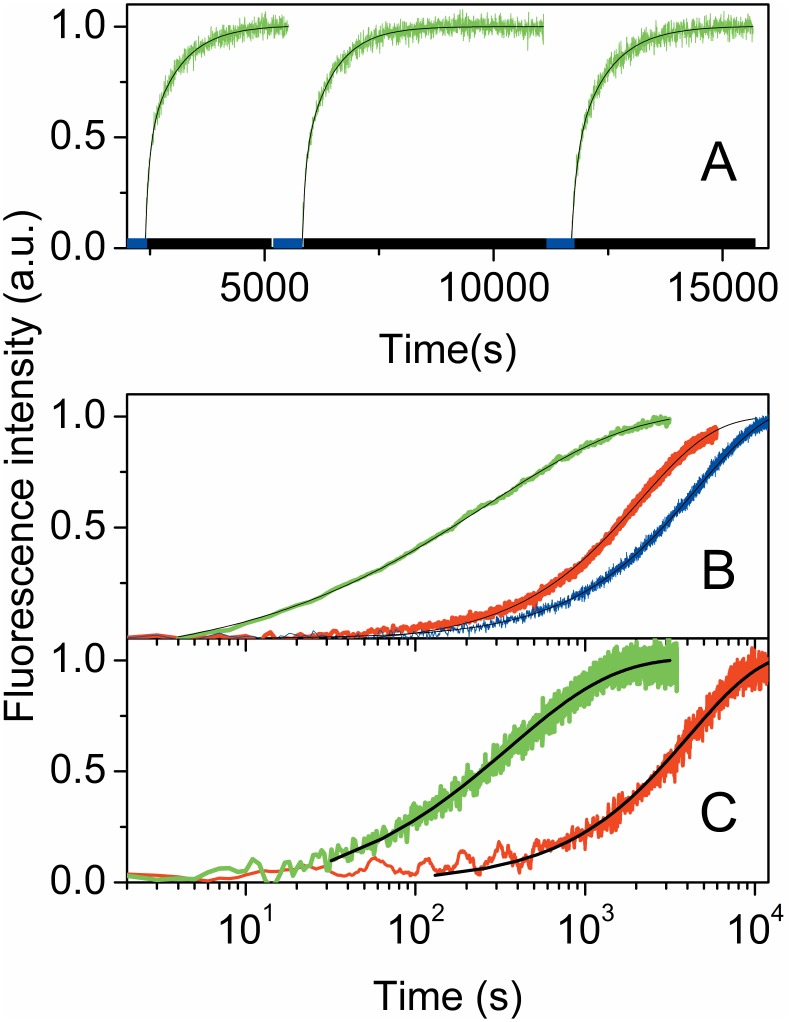
Dark recovery kinetics of YtvAL to YtvAD. **A.** Dark recovery of YtvAL to YtvAD in *E. coli* colonies expressing YtvA deposited on a glass coverslip, after 5 minutes illumination with LED465, monitored through fluorescence emission of YtvAD. The blue bars indicate illumination periods with LED465, black bars indicate that colonies were kept in the dark. T = 25°C. **B.** Dark relaxation of YtvAL to YtvAD, as monitored by the recovery of fluorescence emission after switching off LED465. Blue, buffered YtvA solution; red, *E. coli* colonies expressing YtvA soaked in 10 mM phosphate buffer solution, containing 0.9% NaCl (W/V), pH = 7.4; green, *E. coli* colonies expressing YtvA smeared on a glass coverslip. T = 25°C. Black solid lines correspond to fits with a stretched exponential relaxation (*E. coli* colonies expressing YtvA smeared on a glass coverslip) or with an exponential decay (YtvA solution and bacteria soaked in a buffer). **C.** Dark relaxation of YtvAL to YtvAD after 5 minutes illumination with LED465 of *B. subtilis* colonies expressing YtvA. Green curves were measured for colonies smeared on a glass plate, red curves were obtained for colonies soaked in an isotonic buffer. Black curves are the best fits with an exponential decay (colonies soaked in a buffer) or a stretched exponential decay (colonies smeared on a glass plate). Fitting parameters are reported in Table 1.

*E. coli* cells overexpressing YtvA were withdrawn from the Petri dish and placed on a microscope glass plate. By this preparation, the cells were exposed to air and kept in the dark for 1 hour. Irradiation of cells, prepared in this manner (i.e., fully adapted in its YtvAD state) by blue light (LED465), leads to reduction of the fluorescence emission from YtvAD due to the formation of the non-fluorescent adduct state YtvAL. When the blue light was turned off and cells were kept in the dark, fluorescence emission intensity increased with time, as a consequence of the thermal relaxation to YtvAD. This process is similar to the well characterized behavior of the purified protein, kept in buffer of given pH and salt concentration. Figure 1A shows several subsequent dark relaxation kinetics following illumination cycles of the cell culture (duration 5 minutes, blue bars at the bottom of Fig. 1A). In these experiments it was not possible to monitor the (forward) photoconversion of YtvAD to YtvAL upon LED465 illumination since the detection system was saturated by the fluorescence emission excited by the intense actinic beam. The kinetics of the dark relaxation is quite reproducible from cycle to cycle, with some loss (∼10%) in the fluorescence emission level attained upon recovery of YtvAD. It remains to be established whether this is due to some partial irreversible bleaching of the photoreceptor or to lack of complete recovery on the accessible time scale of our experiment, which is difficult to extend beyond ∼10^4^ s. Control experiments on *E. coli* colonies expressing the C62S YtvA mutant, for which the photocycle is inhibited, showed no modulation of the fluorescence intensity upon illumination (data not shown), thus demonstrating that the observed cyclic effect is indeed related to the photochemistry of the expressed photoreceptor. As a further control, the fluorescence emission intensity from *E. coli* cultures, which were not transformed to express YtvA, was detected and found to be at least two orders of magnitude smaller than the level observed for transformed bacteria.

The first striking feature of the kinetics in Figure 1A is that under these conditions the relaxation becomes much faster than expected on the basis of previous experiments on purified proteins in solution. Under such conditions, in homogeneous solution YtvAL relaxes to YtvAD with an exponential decay characterized by a lifetime of about 4000 s at 25°C. [1] This difference can be appreciated in Figure 1B, which compares the relaxation kinetics observed for the colonies smeared on a glass plate and exposed to air (green curve) and for the purified protein in solution (blue curve). Furthermore, for YtvA molecules within *E. coli* cells the dark relaxation is best described by a stretched exponential decay of the form F = F_0_ exp(−(t/τ)^β^), whose small time constant τ and the smaller than 1 stretching exponent β, reported in Table 1, suggest that YtvAL is much less stabilized than for the purified protein in solution, and that kinetics is stretched in time.

**Table 1:** Apparent time constants for the YtvAL to YtvAD relaxation at 25°C.

	YtvA	τ_d_ (s)	β_d_	τ_w_ (s)	β_w_	γ = τ_w_/τ_d_
		Dry Cells		Wet Cells		
*B. subtilis*	*wt*	360±10	0.70±0.05	4080±40	1	11
*E. coli*	*wt*	263±3	0.51±0.01	2100±10	1	8.0
	R63K	275±38	0.56±0.05	312±12	0.94±0.05	1.1
	N37C	286±5	0.6±0.1	912±2	0.811±0.002	3.2
	T30S	485±46	0.59±0.01	1209±2	0.991±0.003	2.5
	T30A	351±28	0.62±0.06	592±59	1	1.7
	N94A	80±30	0.5±0.1	120±30	1	1.5
	N104D	290±20	0.66±0.04	1100±200	1.05±0.03	3.8
	N104A	140±40	0.5±0.1	620±10	0.98±0.02	4.4
	Q123N	64±2	0.56±0.05	74±7	1	1.1
	Q66H	242±32	0.64±0.03	753±56	0.98±0.02	3.1

When, instead, *E. coli* colonies were placed inside a microscope well and soaked in a physiological buffer (red curve in Figure 1B), thus protecting against de-hydration, the kinetics becomes much slower and are comparable to those observed for purified proteins in solution. In addition, the kinetics is much less distributed and approaches a pure exponential decay, with lifetime very similar to the one observed for YtvA in buffered solution.

Similar effects are observed when the dark relaxation of YtvAL to YtvAD is followed for *B. subtilis* colonies transformed to overexpress the photoreceptor from an encoding plasmid while the gene for YtvA was silenced (Figure 1C).

**Figure 2:**
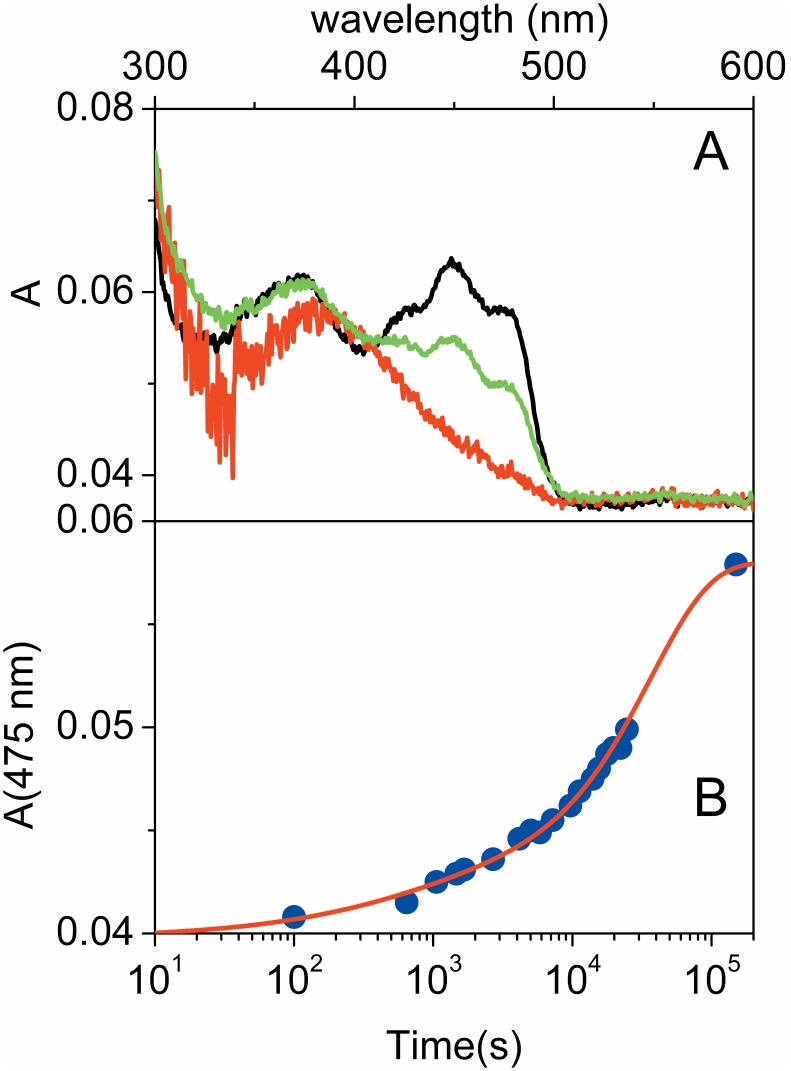
Dark recovery kinetics of dried YtvA. **A.** Absorption spectrum for YtvA molecules air-dried on a quartz plate before (black) and after (red) photoconversion with LED465. The green curve reports the absorption spectrum at t = 24660 s at 25°C. After 24 hours the YtvAD spectrum is fully recovered (not shown). **B.** Dark recovery kinetics of YtvAL to YtvAD followed through the absorbance at 475 nm. The red solid line is the best fit with a double stretched exponential relaxation, with τ = 600±400 s and β = 0.6±0.2 (16%) and τ = (3.7±0.3)×10^4 ^s and β = 1.0±0.2 (84%).

The above experiments suggest a major effect of the cellular environment on the stabilization of YtvAD, which appears to be strongly modulated by the water content of the system. However, when we monitored the recovery of the absorption spectrum of YtvA molecules dried on a glass plate, subject to illumination with LED465 and subsequently left in the dark, we observed a much more complex behavior, which suggests that hydration alone may not account for the intracellular response of the photoreceptor. As shown in Figure 2, after a fast phase accounting for nearly 20% of the absorption which is best described by a stretched exponential relaxation, full recovery is observed through a subsequent exponential decay only after about 24 hours at 25°C. A detailed analysis of this behavior is beyond the scope of the present work and will be reported elsewhere.

**Figure 3:**
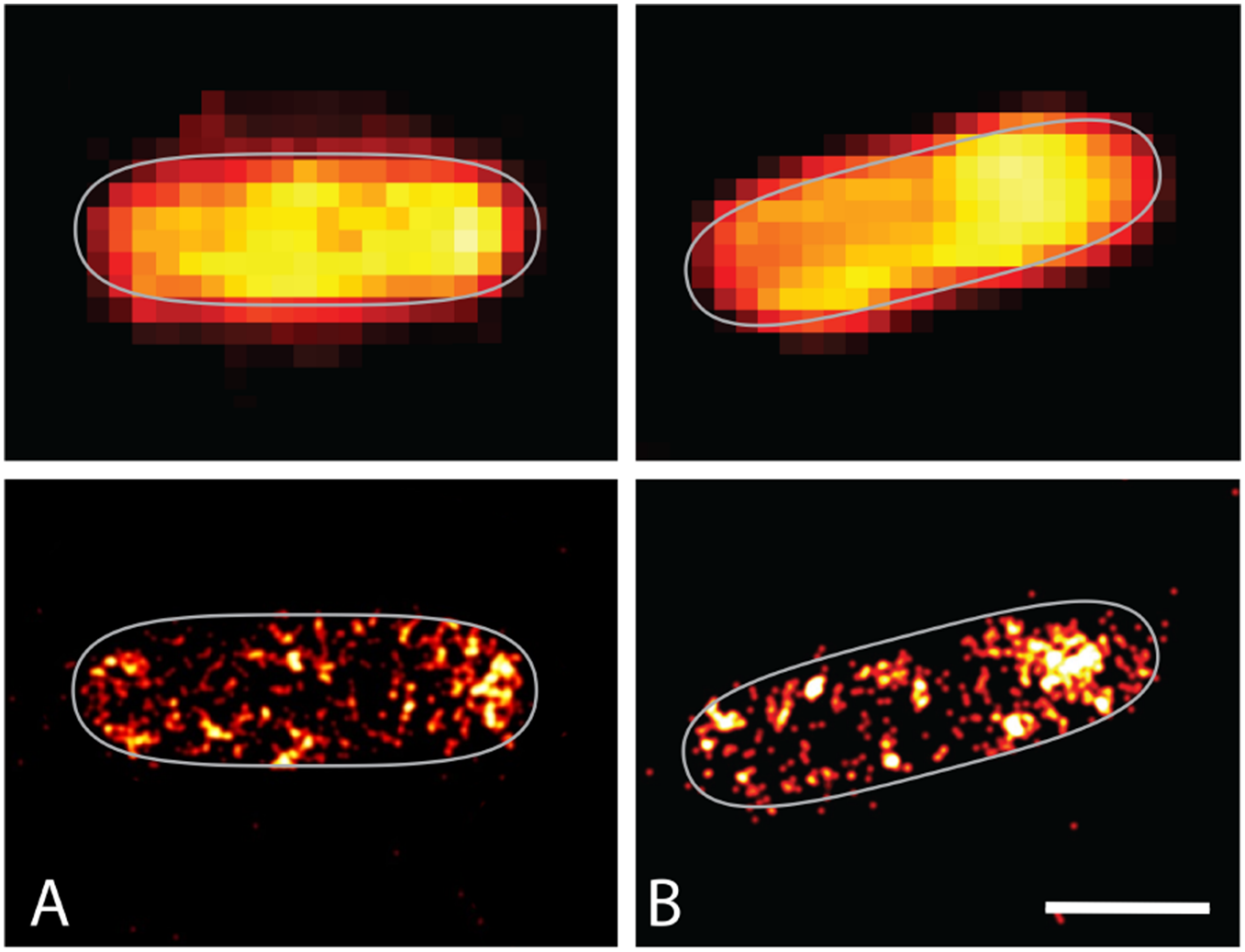
FPALM images of *E. coli* cells expressing wt YtvA. FPALM images of *E. coli* cells expressing wt YtvA soaked in isotonic buffer (A) and under de-hydrated conditions (B). Intracellular distribution seems similar for the system in both hydration states, the protein remains widespread over the cell body with some major aggregates at the cell wall. Experimental conditions for the de-hydrated state: Intensity: 0.2 W/cm^2^ at 405 nm and 0.05 kW/cm^2^ at 488 nm; for the hydrated state: Intensity: 1–2 W/cm^2^ at 405 nm and 0.2 kW/cm^2^ at 488 nm; frame rate: 30 Hz and total number of collected frames: 20000. Scale bar: 1 µm.

The obvious concern for such experiments, i.e., working with the hydrated cells, is that when soaked in a buffer, even if isotonic and thus preventing rupture of cells by trivial osmotic effects, cells may anyway release the photoactive proteins to the surrounding medium and thus expose them to an environment identical to the one experienced by purified proteins in solution. To rule out this possibility we have collected FPALM images of *E. coli* cells over-expressing YtvA, both in their growth medium and soaked in the isotonic buffer employed in the above experiments. Figure 3 compares typical images collected for cells smeared on a glass plate (B) and soaked in isotonic buffer (A). The cellular distribution of the fluorescent proteins is similar in both cases, with accumulation of molecules at the periphery of the cell. Although the background fluorescence emission outside the cells is substantial, the amount of photoswitchable proteins is a negligible fraction, demonstrating that leakage of the expressed YtvA is small. Thus, the amount of water of the environment outside the cells appears to influence proteins located inside the cells.

### Photoconversion studies on YtvA mutants

The so far described empirical correlation between the dark relaxation time constant and level of hydration implies some major degree of coupling between the flavin fluorophore and the surrounding protein environment. The FMN chromophore of YtvA is effectively protected from interactions with solutes as demonstrated by the lack of quenching by molecular oxygen on singlet and triplet excited states, but, on the other hand, it has been proven to be strongly hydrogen-bonded to amino acids in its close vicinity. It is therefore important to identify specific amino acids surrounding the chromophore which for the physiological function transfer the chromophore-generated biological signal to the STAS domain, and thus are capable of transducing the interaction with the solvent. We could previously identify several amino acids nearby the chromophore, which upon mutation caused significant effects on the photocycle rate constants [9], [10].

**Figure 4:**
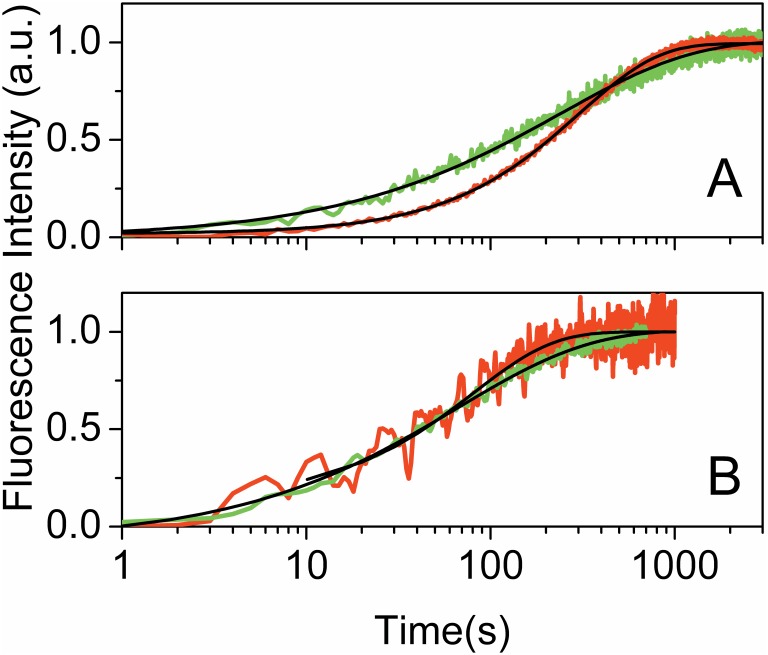
Dark recovery kinetics of *E. coli* colonies expressing selected YtvA mutants. **A.** Dark relaxation of YtvAL to YtvAD after 5 minutes illumination with LED465 of *E. coli* colonies expressing the R63K mutant of YtvA. **B.** Dark relaxation of YtvAL to YtvAD after 5 minutes illumination with LED465 of *E. coli* colonies expressing Q123N YtvA. Green curves were measured for colonies smeared on a glass coverslip, red curves were obtained for colonies soaked in an isotonic buffer. Black curves are the best fits to stretched exponential decays. Parameters are reported in Table 1.

**Figure 5:**
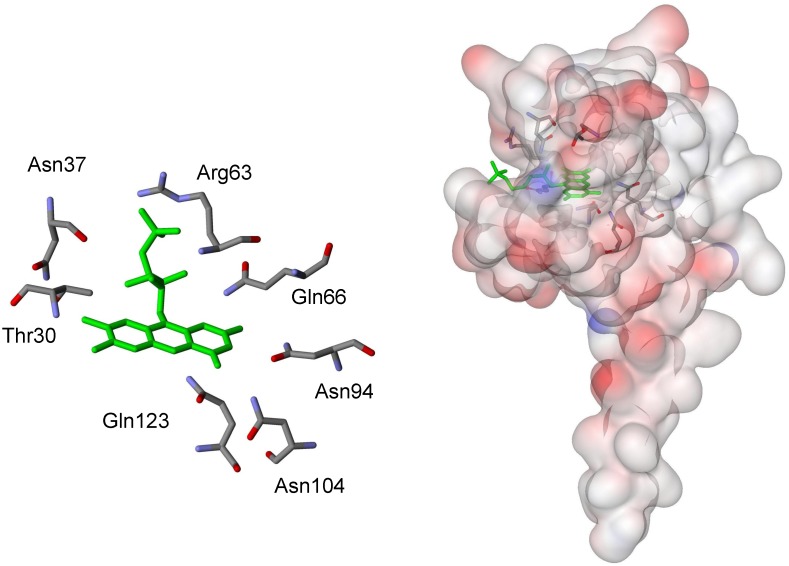
Three dimensional arrangement of mutated amino acids in YtvA. Left. Closeup of the YtvA chromophore (in green) with the amino acids considered in this study represented in capped sticks (red oxygen, blue nitrogen). Right. Solvent accessible surface visualization of the protein with the chromophore in green and the amino acids considered in this study represented in capped sticks.

For a more quantitative comparison between wild type and mutated proteins, we used the ratio **γ** between the time constants measured for bacteria soaked in isotonic buffer (**τ_w_**) and for colonies smeared on a glass plate (**τ_d_**) as an indication of the sensitivity of the dark relaxation to hydration. For wt YtvA, **γ** has a value of about 10 when the dark relaxation is measured either in *B. subtilis* (**γ** = 11) or *E. coli* (**γ** = 8). Figure 4 shows representative relaxation kinetics for *E. coli* colonies expressing selected YtvA mutants, along with the best fits to exponential decays or stretched exponential relaxations (see Table 1 for parameters). While all mutations appear to reduce the value of **γ** in comparison to bacteria expressing the wt protein, specific residues afford values of **γ** close to unity, almost completely removing the effect of the excess solvent in the surrounding medium. Figure 5 shows the topological arrangement of the amino acids surrounding the FMN chromophore considered in this study.

## Discussion

The experimental evidence reported above, demonstrates that the rate of the dark recovery reaction of YtvA is remarkably increased in the cellular environment for colonies smeared on a glass plate. A similar acceleration of the recovery kinetics within cells on agar plates has been reported recently in *E. coli* over-expressing *As*LOV2. [12] In that case, purified *As*LOV2 regains its level of fluorescence after 5 minutes in the dark, with a rate constant of 1.8×10^−2^ s^−1^ at RT, whereas within cells fluorescence is regained within 3 minutes. In the same work, some mutants appear to be extremely sensitive to sample conditions, e.g., the ‘slow cycling’ V416L has a lifetime of 4300 s in vitro and of ca. 230 s within cells, whereas ‘fast cycling’ I427V shows lifetimes of ca. 4 s and 5 s under both conditions, respectively. It should be mentioned that an earlier random mutagenesis work reported that the time scale of fluorescence recovery determined for *As*LOV2-expressing *E. coli* coincided with the rate of adduct decay reported for the purified protein in solution [13].

We similarly observed that, upon increasing the water content of the system by soaking cells in an isotonic buffered solution (Figure 1 and Table 1), the dark recovery rate of the purified protein in solution is restored. This is a clear indication that water plays a major role in controlling the dark relaxation rate. Water content is known to exert major effects on protein functional properties, by strongly affecting their dynamics. In an elegant series of works, Cordone and coworkers have demonstrated that the water content modulates the dynamics of proteins embedded in trehalose glasses. [14], [15], [16], [17] In particular, they showed that the rate of rebinding kinetics of carbon monoxide to ferrous myoglobin after nanosecond laser photolysis is dramatically increased by reducing the amount of residual water, due to the hindered protein motions which prevent escape of the photodissociated ligands from the distal heme pocket. [14], [17], [18] It can be expected that introducing strong perturbations in the bulk solvent structure will influence also those layers of water molecules strictly associated with the protein, which in other examples have been proven to be instrumental for protein dynamics. [17], [19], [20] This sort of perturbation is bound to affect functional events, strongly relying on protein dynamics and on the presence of weak, yet specific stabilizing interactions through hydrogen bonds.

The faster dark recovery observed here for YtvA in cells smeared on a glass plate reflects the existence of a lowered energetic barrier when the protein is exposed to reduced hydration, which appears fundamental in assisting the stabilization of YtvAL. To this end, it is important to recall that an extended hydrogen-bonding network stabilizes the isoalloxazine ring of the flavin mononucleotide chromophore within the photosensing LOV domain of YtvA. The hydrogen bond network includes conserved glutamine and asparagine residues (Figure 5) and has a strong influence on the efficiency, kinetics, and energetics of the protein photocycle. [9] When critical amino acids are mutated, and the hydrogen bonds network is perturbed, observed effects include either increased or strongly reduced rate of adduct formation, and acceleration of the overall photocycle (depending of the amino acid mutated). [9], [10] For mutations N94S, N94A, and Q123N, responsible for breaking strong hydrogen bonds with the chromophore, the dark relaxation occurs with lifetimes 20, 45, and 85 times faster than for wt YtvA [9].

Structural information has suggested the existence of pathways connecting the bulk solvent with the flavin ring in *Nc*VVD (*Neurospora crassa* Vivid) and *As*LOV2. [11], [21] A water gate has been tentatively identified in *As*LOV2 as formed by the N414-Q513 couple (G26 and Q123 in YtvA), whereas C76 and T83 in VVD (corresponding to YtvA T30 and N37) have been proposed to be a water access point. [22] Mutations at C76 in VVD, and the corresponding T418 of *As*LOV2, influence indeed the recovery kinetics, although the effects in the latter protein are much more pronounced than for VVD. [12] VVD C76A, designed to increase solvent accessibility and to decrease steric crowding, accelerated the recovery only about 1.6-fold. [22] A similar increase in the rate constant is observed in *Rhodobacter sphaeroides* LOV, upon T21V change [22].

Mutations at T30 in YtvA are responsible for spectral tuning in the UVA and have an effect on the photocycle kinetics, such that the recovery is accelerated ca. 3.5-fold for T30A/S. [10] This variant is less sensitive than *wt* YtvA to the hydration state of the cells (Table 1). At the same time, the fast cyclic mutant Q123N is almost completely insensitive to this latter parameter, strengthening the case for a water gate close to that position (Table 1) and a participation of this “flipping” glutamine to the proposed base-catalyzed mechanism of proton-abstraction from N5. [23] Imidazole was found to enhance substantially the thermal decay rate of the covalent adduct in several LOV domains, such as *A. sativa* phot1LOV2, *Adiantum* phy3LOV2, and *Chlamydomonas* photLOV2. [24] A general base catalysis mechanism was proposed, [24] in analogy with that suggested for the BLUF domain in AppA from *Rhodobacter sphaeroides.* [25] The effect of imidazole on LOV and BLUF domain-containing photoreceptors is taken as evidence that basic residues, especially histidines, may play an important role in the tuning of the thermal recovery rate of the adduct-containing signaling state. Similarly, imidazole was found to decrease the time constant from 91 minutes to 4.5 minutes for the YtvA-Like *Listeria monocytogenes* protein as a consequence of a reduced activation enthalpy and an increased activation entropy. [26] On the other hand, the presence of tunnels connecting the solvent and internal cavities in the *Avena sativa* Phototropin-1 LOV2 domain was proposed to be instrumental to the observed effect induced by imidazole, by holding the ligand in a position close to that of the chromophore. [24] This topological feature was suggested to be an intrinsic structural property of the PAS fold since similar structures are observed in the crystal structure of CrLOV1, [27] PYP, [28] and NifL [29].

The mutual contribution of solvent and glutamine at that particular position to this rate-limiting step remains nevertheless unclear. Polar substitutions at the corresponding Q513 residue of AsLOV2 accelerate the photocycle while apolar changes slow down the kinetics. [11] This residue is also generally responsible for signal propagation in LOV domains, and in YtvA its substitution with asparagine also impairs light-induced structural changes and diminishes the photocycle quantum yield. [9] Its role is therefore complex and crucial, probably also involving local structural features.

The role of N94 has been poorly investigated in LOV proteins, although this residue is conserved and is part of the extended HB network around the chromophore. [30] Molecular dynamics simulations predict a crucial role for this residue and for N104 (N482 and N482 in AsLOV2) in initiating, responding, and propagating the light-triggered conformational changes. [31] However, chemical-physical studies *in* *vitro* proved difficult for point-mutated *As*LOV2 due to poor expression yield and instability of the protein, given the crucial role of these residues in stabilizing the chromophore by means of an extended HB network. [32] For YtvA, mutations at N104 are not destabilizing the protein but had an only moderate effect on the photocycle dynamics. [9] Changes at N94 (that establishes HBs with C(2) = O, N(1) and Q66) had a much larger influence both on spectral features in the UVA region and on the kinetics. [10] N94A not only shortens the recovery lifetime ca. 40-fold at 20°C, but also increases the triplet lifetime to 129 µs (2 µs in *wt* YtvA). [9] Unfortunately, we cannot compare these results with experimental data from other systems. Nevertheless, recent studies with the EL222 LOV protein from *Erythrobacter litoralis* have revealed that HB interactions at C(2) = O, N(1) and N107 (corresponding to N94) are crucial for determining an Arrhenius behavior for the adduct decay in LOV domains. [33] EL222 bears an alanine in place of a glutamine at position Q66 (A79) that cannot form the local HB network that would involve the corresponding of N94 and induces a non-Arrhenius behavior for adduct decay. A set of mutations demonstrated that this results from increased solvent access and increased flavin vibrational freedom, thus establishing a further water access point. [33] Furthermore, the authors suggested that changes of the HB network also induce local structural changes: this feature might influence not only signal propagation but also the activation energy for the recovery reaction. In this respect, we note that the much shorter recovery kinetics for YtvA-N94A is due to a sharp increase in the activation entropy with respect to *wt* YtvA, i.e. +100 kJ K^−1^ M^−1^ vs +31 kJ K^−1^ M^−1^. Instead, shortening of adduct lifetime in Q123N, T30A and R63K [34] is due to a large decrease in the enthalpic term of the activation barrier. [9], [10] This latter mutation (R63K) is the sole among those having γ close to 1 that does not involve interaction at the isoalloxazine ring. R63 is part of an extended HB network that involves the terminal phosphate of FMN and Asp36 and is directly succeeding the reactive cysteine (C62). [35] Substitution with a lysine is expected to partially destroy this network and to induce local structural changes, possibly by clamping the lysine lateral chain to the ribityl chain of the flavin. The role of this residue has been poorly investigated, but the corresponding mutation in phot-LOV1 from *Chlamydomonas reinhardtii* (R58K) also shortens the recovery lifetime from 200 s to ca. 70 s at 20°C [36].

Given that the rate limiting step of splitting the covalent bond between FMN and protein in the photoadduct is suggested to be base-catalyzed by water, [24], [25] i.e., should be initiated by a proton abstraction from N(5), [37] it is hardly imaginable that the effects of R63K on the recovery kinetics relating to hydration level of cells, rely on this process. In the absence of structural information, we do not have a definite answer but it can be suggested that, at least in this case, conformational/structural effects are predominant. Given that this mutation does not impair protein stability [36] or interdomain signaling, [34] it could be exerted for other LOV proteins, both in vitro and in vivo and also might be considered as an important factor for structural investigations.

The faster dark relaxation of YtvAL to YtvAD observed within *E. coli* cells has profound implications for cellular applications of flavin binding LOV domains. We have recently presented YtvA as a genetically encoded fluorescent probe for super-resolution microscopy based on random activation of single molecules. In particular we reported Fluorescence Photo-Activation Localization Microscopy (FPALM) studies of live *E. coli* cells, expressing YtvA molecules, thus demonstrating the photoswitching properties of YtvA in living cells [5].

In FPALM, molecules are normally kept in their light adapted, non-fluorescent state, and fluorescence from individual molecules is activated by exposing the sample to a suitable laser source of low intensity. In our application we found necessary to apply a continuously running laser source emitting at 488 nm to inhibit build-up of an appreciable population of fluorescent molecules through dark relaxation. [5] This fact was at odds with the very slow dark relaxation observed for YtvA in solution, suggesting that once photoconverted to YtvAL, the molecules should remain in their non-fluorescent state for very long times, inconsistent with the observation of fast recovery of the fluorescent state YtvAD.

The shorter dark recovery time may have consequences for optogenetic applications of actuators based on the flavin binding LOV domains as photosensitive elements, if the physiologically active state confers to a light-induced enzyme activity or similar. Due to the cellular environment, the biologically active, light adapted state will have a shorter persistence and thus allow a faster recovery of the inactive state. This is a relevant property that could be exploited to tailor the recovery kinetics to specific needs, in view of the prospected wide range of optogenetic applications of LOV domains [38], [39], [40].

It is thus worth considering that the observed change in rate constants may be rationalized also in a different perspective. When co-solutes are present at high concentrations, as inside a living cell, the volume available for diffusion of reactants participating in a chemical reaction is dramatically reduced due to the mutual impenetrability of all solute molecules. This effect is often referred to as molecular crowding. [41], [42], [43] Effects of crowding on biochemical reaction rates are complex, because although crowding reduces diffusion, it increases thermodynamic activities. For a transition-state-limited reaction, crowding is expected to increase the rate constant because crowding increases the activity. This argument appears relevant also in view of our previous FPALM studies on live *E. coli* cells overexpressing YtvA, demonstrating preferential allocation of the protein in the membrane regions of the bacteria. [5] At present it is unclear whether this location is due to its interactions with proteins similar to those naturally occurring in the stressosome of *B. subtilis*, or to artifactual accumulation inside inclusion bodies. In any event, a dense packing into multicomponent assemblies is expected to lead to substantial molecular crowding effect which may account for the observed changes in the measured rate constants.

Further studies under strictly controlled water content conditions on purified proteins and in the presence of crowding agents will yield a more quantitative connection between the observed effect and hydration of the protein. Furthermore, a possible role of the specific ions in the salts used to stabilize the pH and define the ionic strength cannot be ruled out at this stage and will be also the subject of future investigations.

The complex kinetics observed for purified YtvA proteins dried on a glass plate with respect to buffer solutions can be compared to the results obtained with *As*LOV2 under similar conditions, for which removal of water causes a significant increase in the recovery lifetime, raising from 80 s to 1726 s at 22°C. [11] For this system, several lines of evidence have suggested that formation of the adduct induces a conformational change concomitant with an increased solvent accessibility, and that water may catalyze N5 deprotonation and splitting of the covalent bond. Nevertheless this might not be the sole effect of water on LOV domain dynamics, as it has been observed that de-hydration impairs the large light-induced conformational changes centered on the β-sheet of the LOV core. [23] FTIR studies demonstrated that light-induced conformational changes of the LOV core seem to be sequential after formation of the adduct, with one intermediate that could be trapped at low hydration levels. [23] Catalytic activity of water on covalent bond splitting and water-assisted conformational changes during the photocycle might exert opposite effects on the recovery kinetics, in that a conformational intermediate trapped at low hydration levels would most probably have a higher energy content, thus lowering the activation barrier. Further studies will aim at characterizing the light-induced protein conformational changes of YtvA at different levels of hydration.
